# A Novel Prognostic Prediction Model for Colorectal Cancer Based on Nine Autophagy-Related Long Noncoding RNAs

**DOI:** 10.3389/fonc.2021.613949

**Published:** 2021-10-08

**Authors:** Guoqiang Xu, Mei Yang, Qiaoli Wang, Liufang Zhao, Sijin Zhu, Lixiu Zhu, Tianrui Xu, Ruixue Cao, Cheng Li, Qiuyan Liu, Wei Xiong, Yan Su, Jian Dong

**Affiliations:** ^1^ Department of Radiotherapy, Yunnan Cancer Hospital, the Third Affiliated Hospital of Kunming Medical University, Kunming, China; ^2^ Cadre Medical Department, Yunnan Cancer Hospital, the Third Affiliated Hospital of Kunming Medical University, Kunming, China; ^3^ The First Department of Head and Neck Surgery, Yunnan Cancer Hospital, the Third Affiliated Hospital of Kunming Medical University, Kunming, China; ^4^ Department of Oncology, Affiliated Hospital of Panzhihua University, Panzhihua Integrated Traditional Chinese and Western Medicine Hospital, Panzhihua, China; ^5^ Department of Graduate Student Management, Yunnan Cancer Hospital, the Third Affiliated Hospital of Kunming Medical University, Kunming, China; ^6^ Department of Medical Oncology, Yunnan Cancer Hospital, the Third Affiliated Hospital of Kunming Medical University, Kunming, China

**Keywords:** colorectal cancer, autophagy, long noncoding RNAs, risk score, prognostic prediction model

## Abstract

**Introduction:**

Colorectal cancer (CRC) is the most common gastrointestinal cancer and has a low overall survival rate. Tumor–node–metastasis staging alone is insufficient to predict patient prognosis. Autophagy and long noncoding RNAs play important roles in regulating the biological behavior of CRC. Therefore, establishing an autophagy-related lncRNA (ARlncRNA)-based bioinformatics model is important for predicting survival and facilitating clinical treatment.

**Methods:**

CRC data were retrieved from The Cancer Genome Atlas. The database was randomly divided into train set and validation set; then, univariate and multivariate Cox regression analyses were performed to screen prognosis-related ARlncRNAs for prediction model construction. Interactive network and Sankey diagrams of ARlncRNAs and messenger RNAs were plotted. We analyzed the survival rate of high- and low-risk patients and plotted survival curves and determined whether the risk score was an independent predictor of CRC. Receiver operating characteristic curves were used to evaluate model sensitivity and specificity. Then, the expression level of lncRNA was detected by quantitative real-time polymerase chain reaction, and the location of lncRNA was observed by fluorescence *in situ* hybridization. Additionally, the protein expression was detected by Western blot.

**Results:**

A prognostic prediction model of CRC was built based on nine ARlncRNAs (*NKILA*, *LINC00174*, *AC008760.1*, *LINC02041*, *PCAT6*, *AC156455.1*, *LINC01503*, *LINC00957*, and *CD27-AS1*). The 5-year overall survival rate was significantly lower in the high-risk group than in the low-risk group among train set, validation set, and all patients (all p < 0.001). The model had high sensitivity and accuracy in predicting the 1-year overall survival rate (area under the curve = 0.717). The prediction model risk score was an independent predictor of CRC. *LINC00174* and *NKILA* were expressed in the nucleus and cytoplasm of normal colonic epithelial cell line NCM460 and colorectal cancer cell lines HT29. Additionally, *LINC00174* and *NKILA* were overexpressed in HT29 compared with NCM460. After autophagy activation, *LINCC00174* expression was significantly downregulated both in NCM460 and HT29, while *NKILA* expression was significantly increased.

**Conclusion:**

The new ARlncRNA-based model predicts CRC patient prognosis and provides new research ideas regarding potential mechanisms regulating the biological behavior of CRC. ARlncRNAs may play important roles in personalized cancer treatment.

## 1 Introduction

Colorectal cancer (CRC) is a common gastrointestinal cancer. According to the 2020 global cancer statistics, more than 1.9 million new cases of CRC were diagnosed, and 935,000 CRC patients died. CRC ranks third in morbidity and second in mortality for all tumors (1). With advancements in comprehensive therapy, including surgery, chemotherapy, biological immunotherapy, and radiotherapy, CRC outcomes are improving, but the overall survival rate is still low (2). Historically, the tumor–node–metastasis (TNM) staging system has been widely used to predict the prognosis of CRC patients. Generally, the prognosis is better with earlier staging. In recent years, however, some studies have shown that the combination of biomarkers and TNM staging is more accurate in predicting the prognosis of CRC patients (3, 4).

Long noncoding RNAs (lncRNAs) are defined as RNAs that contain more than 200 nucleotides and do not encode a protein. They play important roles in transcription, translation, and cell cycle regulation (5). A growing body of evidence indicates that lncRNAs play important roles in CRC occurrence, development, metastasis, and drug resistance (6–9). Moreover, several lncRNA-based prediction models have been built to predict the survival of patients with CRC (10–12), indicating that lncRNAs can serve as CRC biomarkers.

Autophagy is a process of intracellular degradation that helps maintain homeostasis by promoting nutrient recycling during nutrient deficiency, hypoxia, DNA damage, and infection (13, 14). It plays an important role in tumor development, maintenance, and progression (15). Studies have shown that autophagy is a double-edged sword, as it promotes CRC invasion and metastasis and CRC cell apoptosis (16, 17). Current studies show that tumor autophagy can predict patient prognosis (18, 19).

Overall, autophagy and lncRNAs both play important biological roles in CRC. To date, few studies have been conducted that investigate the role of autophagy-related lncRNAs (ARlncRNAs) in the survival of cancer patients. One study showed that the prognosis of patients with lung adenocarcinoma is related to the abnormal expression of 13 ARlncRNAs (20). We hypothesize that ARlncRNAs are closely related to the survival of CRC patients and that ARlncRNAs are potential biomarkers of and treatment targets for CRC. In this study, using bioinformatics technology, we identified and screened ARlncRNAs related to the prognosis of CRC patients and built a novel model that can be used to predict prognosis. This model is of great significance in predicting the prognosis of CRC patients in clinical practice.

## 2 Methods

### 2.1 Data Collection

We downloaded transcriptome and clinical data of CRC samples from The Cancer Genome Atlas (TCGA: https://portal.gdc.cancer.gov/repository) database. RNAs were annotated *via* human gene annotation files (GRCh38.p12) downloaded from the Ensembl database (https://asia.ensembl.org/index.html). Next, we downloaded human autophagy-related gene profiles from the Human Autophagy database (HADb: http://www.autophagy.lu/clustering/index.html).

### 2.2 Data Analysis

**Figure 1:**
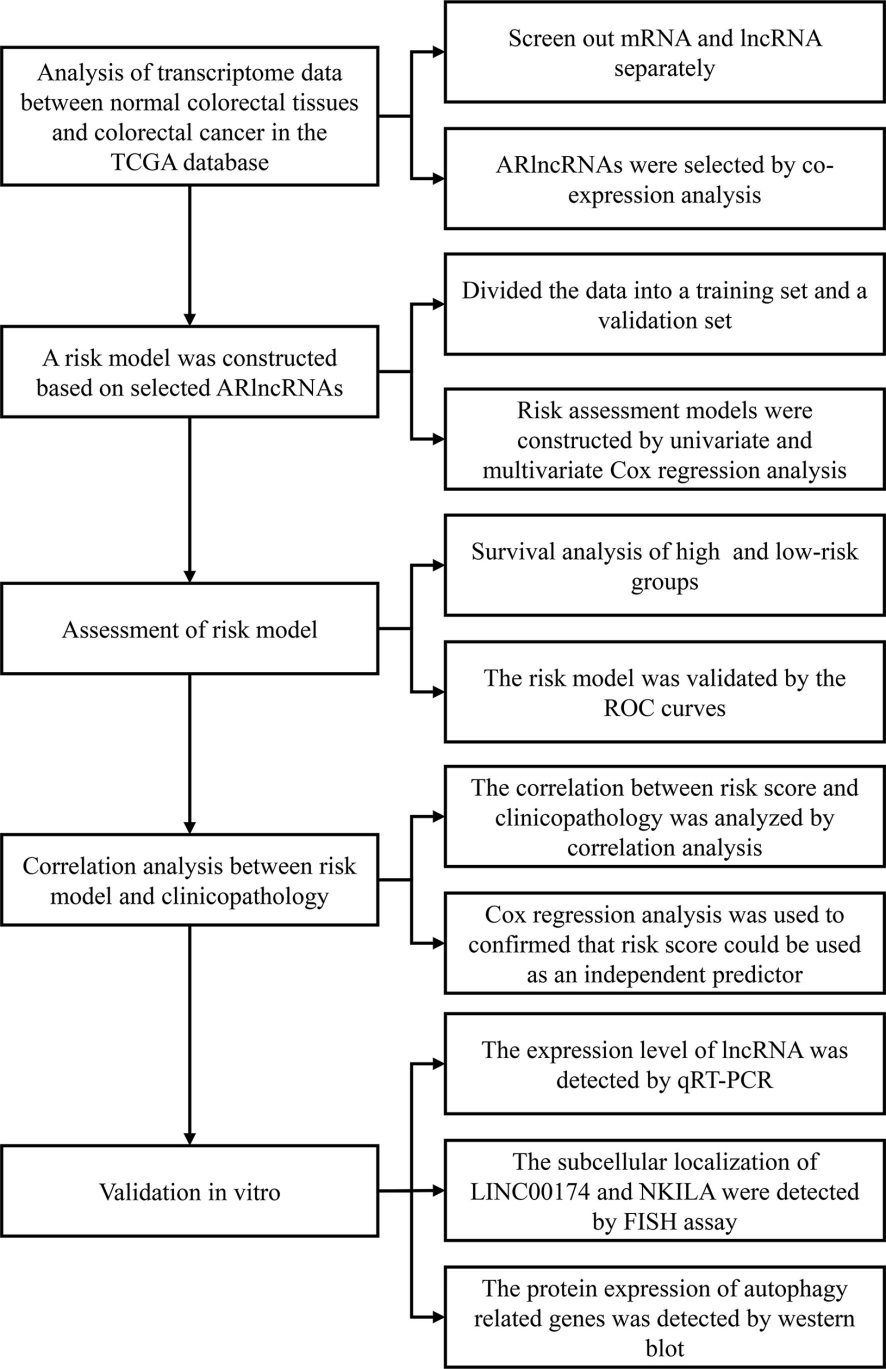
The flow chart of overall procedures. TGCA, the Cancer Genome Atlas; IncRNA, long non-coding RNA; ARIcRNAs, autophagy-related IncRNAs; ROC, receiver operating characteristic; qRT-PCR, quantitative real-time polymerase chain reaction; FISH, fluorescence in situ hybirdization.

We employed Strawberry Perl software (v5.30.2.1) to compile the clinical data of CRC patients. After deleting “unknown” and incomplete survival data (“NULL”), we recorded the survival time, survival status, age at diagnosis, sex, clinical stage, T stage, M stage, and N stage of each patient. We used the Strawberry Perl program to organize the transcriptome data of CRC samples into an expression matrix and then used GRCh38.p12 to annotate the genes. Next, we obtained separate messenger RNA (mRNA) and lncRNA expression matrices for CRC samples and used the “limma” package in R (v4.0.0) for coexpression analysis of the mRNA expression matrix and autophagy-related gene profile to obtain the expression matrix for autophagy-related genes. Then, we performed coexpression analysis of the autophagy-related expression matrix and lncRNA expression matrix (selection criterion: absolute correlation coefficient > 0.3, p < 0.001) to obtain the lncRNA matrix coexpressed with autophagy-related genes, that is, ARlncRNAs. We utilized Strawberry Perl software to merge the clinical data of CRC patients and the information from the ARlncRNA expression matrix to obtain a matrix of the expression levels of ARlncRNAs and survival status. The flow chart of overall procedures is shown in **Figure 1**.

### 2.3 Model Establishment

We used the “caret” package in R to divide the database into train set and validation set. Then, we used the “survival” package in R to conduct univariate Cox regression analysis (p < 0.05) to screen ARlncRNAs related to the 5-year OS of CRC patients and then performed multivariate Cox regression analysis with optimization based on the optimal Akaike information criterion to screen ARlncRNAs for the prediction model. The risk score was the sum of the product of the expression level of each ARlncRNA and the corresponding multivariate Cox regression coefficient (21, 22). The above model was applied to calculate the risk score for each patient. The patients were divided into high- and a low-risk groups with the median risk score as the cutoff value. Next, the “pheatmap” package in R was employed to plot the heat maps of nine ARlncRNAs in each group.

### 2.4 Survival Analysis

We used the “survival” package in R to plot Kaplan–Meier survival curves to analyze the 5-year OS associated with high or low expression of each ARlncRNA and then performed the log-rank sum test to analyze survival differences between the high and low expression groups. We also compared the 5-year OS between the high-risk group and the low-risk group obtained from this model. Next, the “pheatmap” package in R was used to plot the survival time and survival status.

### 2.5 Model Evaluation

We utilized the “survivalROC” package in R to plot the receiver operating characteristic (ROC) curve to evaluate the sensitivity and specificity of the ARlncRNA-based risk model *via* the area under the curve (AUC). Moreover, we analyzed the performance of the risk score from the ARlncRNA-based model *versus* TNM stage, age, sex, and clinical stage for predicting 5-year survival. In addition, we performed multivariate Cox regression analysis and stratified analysis to determine whether the risk score was independent of clinical variables.

### 2.6 Gene Ontology and Kyoto Encyclopedia of Genes and Genomes

Gene Ontology (GO) and Kyoto Encyclopedia of Genes and Genomes (KEGG) analyzed the signal pathways of enrichment of genes that construct an interaction network with lncRNAs in the model.

### 2.7 ARlncRNA Interactive Networks

We used Perl software to screen autophagy-related genes coexpressed with prognostic ARlncRNAs and then used Cytoscape software (v3.8.0) to plot and visualize interactive networks of ARlncRNAs and mRNAs from the prediction model. Next, we utilized the “ggplot2,” “gglluvial,” and “dplayr” packages in R to plot a Sankey diagram to determine whether the ARlncRNAs included in the model were risk factors or protective factors.

### 2.8 Validation *In Vitro*

#### 2.8.1 Cell Culture

Human colorectal cancer cell lines HT29 (Procell Life Science & Technology Co., Ltd., China), HCT116, and RKO (BeNa culture collection, China) and normal colonic epithelial cell line NCM460 (BeNa culture collection, China) were used for further investigations. Cell line HCT116 was cultured in Roswell Park Memorial Institute (RPMI) 1640 complete medium containing 10% fetal bovine serum (FBS), RKO, and NCM460 in Dulbecco’s modified Eagle’s medium (DMEM) (H) complete medium containing 10% FBS, and cell line HT29 in McCoy’s 5A complete medium containing 10% FBS. The cells were cultured at 37°C in an incubator and passaged by 0.1% trypsin digestion every 3–4 days during the logarithmic growth period. All cells were grown addictively. To detect the function of lncRNAs in autophagy, cells were applied with 25 nM mammalian target of rapamycin (mTOR) inhibitor Rapamycin for 72 h for further investigation.

#### 2.8.2 Quantitative Real-Time Polymerase Chain Reaction

RNAs were extracted using the Trizol reagent (Invitrogen, Carlsbad, CA, USA), followed by removal of DNA with the TurboDNase kit (Ambion). Quantification of extracted RNA was performed using NanoDrop. Complementary DNA synthesis was performed using PrimeScript real-time (RT) reagent kit (Takara Bio, Japan) using 1,000 ng of total RNA. Quantitative real-time polymerase chain reaction (qRT-PCR) was performed using the SYBR Select Master Mix (Applied Biosystems, Waltham, MA, USA) on an ABI 7900 system (Applied Biosystems). Glyceraldehyde 3-phosphate dehydrogenase (GAPDH) was used as a control. The Ct value was calculated based on the ΔΔCt method. Fold change of gene expression was expressed as 2^−ΔΔCt^. The primers used in this study were as follows: *NKILA*, sense strand 5′-CGGATACATCTTAGTTGTTATG-3′, antisense strand 5′-GTGCTGGAATCATCATTG-3′ and *LINC00174*, sense strand 5′-GCATTAGATTCTCATAGG-3′, antisense strand 5′-GGCATTAGATTCTCATAG-3′.

#### 2.8.3 Western Blot

*LC3B*, *p62*, *beclin1*, and *ATG7* were extracted from the indicated cells using radioimmunoprecipitation assay (RIPA) lysis buffer, and a BCA Protein Assay Kit (Thermo Scientific, USA) was used to measure the protein concentration. In total, 60 μg of protein was separated on 10% sodium dodecyl sulfate–polyacrylamide gel electrophoresis (SDS-PAGE) gels by PAGE and transferred onto nitrocellulose membranes. The membranes were blocked using 5% non-fat dry milk and incubated with primary rabbit monoclonal antibody overnight at 4°C. The membranes were washed with Tris-buffered saline with Tween 20 (TBST) and then incubated with the appropriate secondary antibody. Enhanced chemiluminescence reagent was used to detect the signal on the membrane. The antibody used in this study are shown in **Supplementary Table 1**.

#### 2.8.4 Fluorescence *In Situ* Hybridization

The subcellular localization of *LINC00174* and *NKILA* was detected by FISH assay. Cells were fixed with 4% paraformaldehyde (Aladdin, China) and incubated overnight at 37°C with a labeled *LINC00174* or *NKILA* probe (Sangon, China). Then, cells were incubated with 4′,6-diamidino-2-phenylindole (Best-Bio, Shanghai, China). The image was acquired by laser scanning confocal microscopy (LSM710, Carl Zeiss, Germany).

### 2.9 Statistical Analysis

The Cox proportional hazard model was employed for univariate and multivariate analyses. The Kaplan–Meier method was used to plot survival curves, and the log-rank sum test was performed to analyze between-group differences. Due to homogeneity of variance, Student’s t-test and Dunnett’s multiple comparisons test in analysis of variance were used to analyze the expression levels of lncRNA in different cells. Statistical analyses were conducted with R (v4.0.0) and GraphPad Prism (v9.2.0) software. A two-tailed value of p < 0.05 was considered statistically significant, unless otherwise specified.

## 3 Results

### 3.1 Identification of Nine Prognostic ARlncRNAs

**Figure 2:**
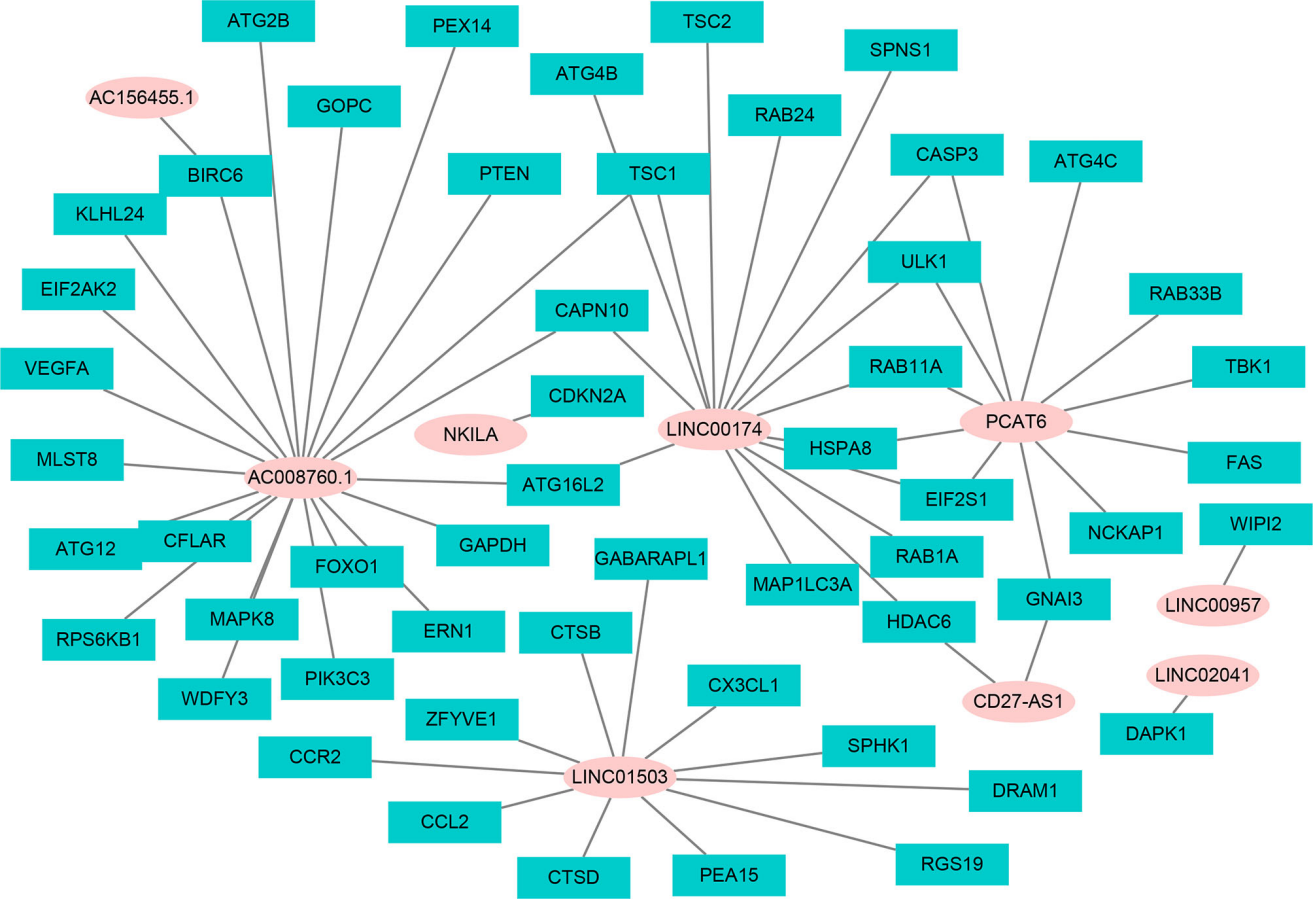
The interaction network of OS-associated lncRNAs and autophagy genes. The pink circle refers to ARlncRNA, and the cyan square refers to autophagy genes.

**Figure 3:**
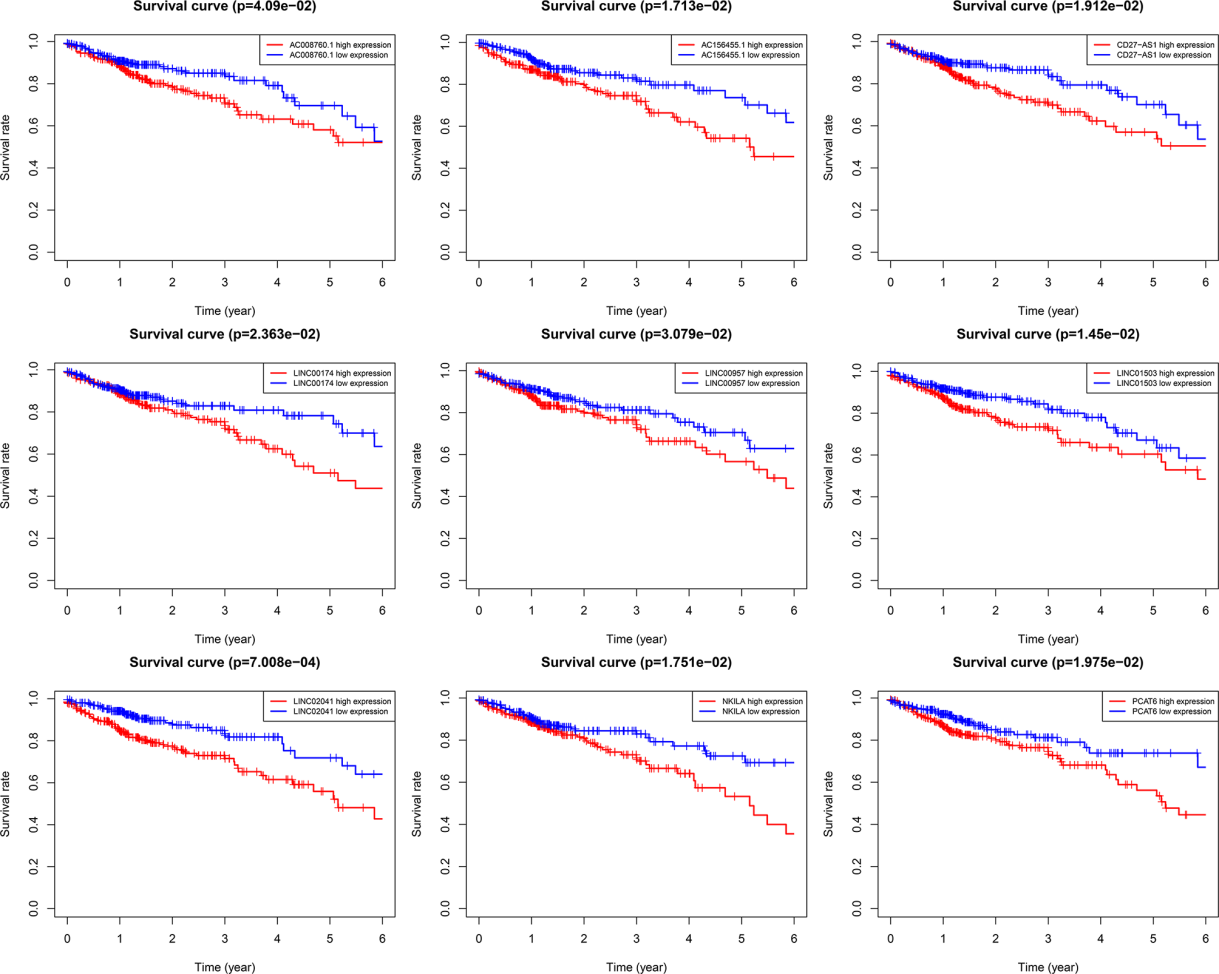
Kaplan–Meier 5-year overall survival (OS) curves for two groups divided by high and low expression level of ARlnRNAs. The red curves correspond to patients with high expression level of ARlnRNAs, while the blue curves correspond to patients with low expression level of ARlnRNAs.

Using univariate Cox regression analysis and the Kaplan–Meier method, we screened 32 prognostic ARlncRNAs (**Supplementary Table 2**). Further multivariate Cox regression analysis of these 32 ARlncRNAs with optimization at an Akaike information criterion of 365.59 yielded an interactive network diagram with 9 ARlncRNAs and 53 coexpressed mRNAs (**Figure 2**). We then plotted Kaplan–Meier survival curves to analyze survival and plotted the corresponding survival curve associated with high or low expression of each of the nine ARlncRNAs (**Figure 3**). The results showed that the 5-year survival rate was significantly lower in the high- than in the low-expression group among all ARlncRNAs (*NKILA*, *LINC00174*, *AC008760.1*, *LINC02041*, *PCAT6*, *AC156455.1*, *LINC01503*, *LINC00957*, and CD27-AS1, all p < 0.05).

### 3.2 Risk Score Model Based on Nine ARlncRNAs

We constructed a proportional hazards model of these nine ARlncRNAs using multivariate Cox regression analysis: risk score = (0.667 × the expression level of *NKILA*) + (−0.601 × the expression level of *LINC00174*) + (1.052 × the expression level of *AC008760.1*) + (0.543 × the expression level of *LINC02041*) + (0.533 × the expression level of *PCAT6*) + (0.843 × the expression level of *AC156455.1*) + (0.950 × the expression level of *LINC01503*) + (0.578 × the expression level of *LINC00957*) + (0.925 × the expression level of *CD27-AS1*) (**Table 1**).

**Table 1:** ARlncRNAs applied to new prediction model.

lncRNA	Coef	HR	95% CI	p-value
NKILA	0.667	1.949	1.200–3.165	0.007
LINC00174	−0.601	0.548	0.237–1.267	0.160
AC008760.1	1.052	2.864	1.081–7.583	0.034
LINC02041	0.543	1.722	1.145–2.589	0.009
PCAT6	0.533	1.704	0.959–3.030	0.069
AC156455.1	0.843	2.323	1.496–3.605	0.000
LINC01503	0.95	2.587	1.475–4.536	0.001
LINC00957	0.578	1.783	0.833–3.816	0.136
CD27-AS1	0.925	2.521	1.342–4.736	0.004

HR, hazard radio; CI, confidence interval.

### 3.3 Evaluation of the ARlncRNA-Based Prediction Model for CRC

**Figure 4:**
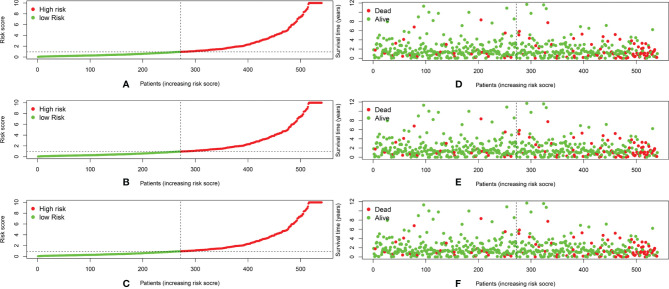
**(A–C)** The distribution of the risk scores of the patients in both high- and low-risk score groups among train set, validation set, and all patients. **(D–F)** Patients’ survival status and time distributed by risk score among train set, validation set, and all patients.

**Figure 5:**
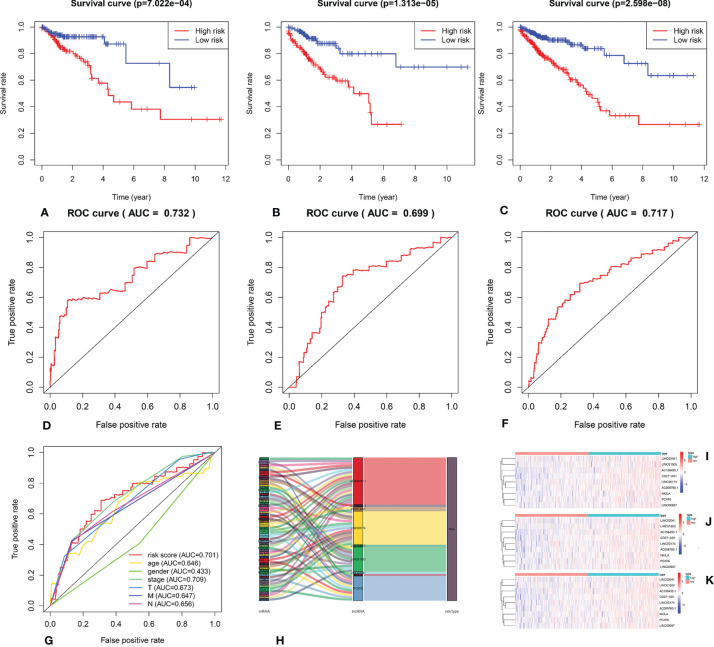
**(A–C)** Kaplan–Meier survival curve of the low- and high-risk groups based on median risk score valued by ARlnRNAs model among train set, validation set, and all patients. **(D–F)** The receiver operating characteristic (ROC) curve of nine ARlncRNAs model among train set, validation set, and all patients. **(G)** The receiver operating characteristic (ROC) curve of nine ARlncRNA risk score and clinicopathological parameters (age, gender, clinical stage, and TNM stage). **(H)** The Sankey diagram shows the connection degree between the 53 mRNAs and 9 ARlncRNAs. **(I–K)** The heatmap of the nine ARlncRNA expression value in low- and high-risk score groups among train set, validation set, and all patients. Red to blue indicates a trend from high to low expression.

We calculated the risk score for each patient based on the proportional hazard model for nine ARlncRNAs and then sorted the risk scores in ascending order. With the median as the cutoff value, we divided the patients into a high- and a low-risk group between train set and validation set. **Figure 4** shows the risk score and survival status. Kaplan–Meier survival analysis indicated that the 5-year OS was significantly higher in the low- than in the high-risk group among train set **(** **Figure 5A**), validation set (**Figure 5B**), and all patients (**Figure 5C**). Moreover, the model had high sensitivity and specificity in predicting the 1-year OS of CRC patients among train set (**Figure 5D**), validation set (**Figure 5E**), and all patients (AUC = 0.717, **Figure 5F**). In addition, multivariate analysis showed that risk value was better than clinicopathological factors (**Figure 5G**). Sankey diagrams and heat maps were used to visualize the expression profiles for the nine ARlncRNAs in the low- and high-risk groups. The results showed that the expression level of all ARlncRNAs tended to be higher in the low- than in the high-risk group, and they were determined to be risk factors (**Figures 5H**–**K**).

### 3.4 Risk Factors for Predicting 5-Year OS

**Figure 6:**
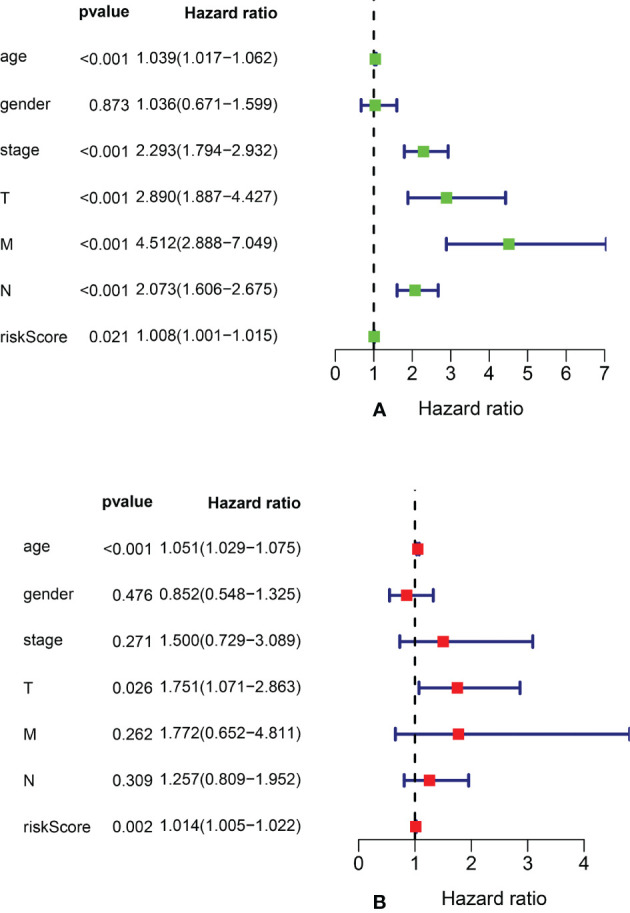
Forest plots of univariate and multivariate Cox regression analyses of clinicopathological parameters (age, gender, clinical stage, and TNM stage) and risk scores associated with 5-year overall survival. **(A)** Forest plot of univariate Cox regression analysis. **(B)** Forest plot of multivariate Cox regression analysis.

We performed univariate Cox regression analysis to screen prognostic clinicopathological factors and then performed multivariate Cox regression analysis to analyze the effect of multiple clinicopathological factors (including age at diagnosis, sex, TNM staging, clinical stage, and risk score from the ARlncRNA model) on 5-year OS to screen independent predictors of 5-year OS. The results showed that sex, clinical stage, N stage, and M stage were unrelated to 5-year OS (p > 0.05), whereas age (HR = 1.051; 95% CI, 1.029−1.075; p < 0.001), T stage (HR = 1.751; 95% CI, 1.071−2.863; p = 0.026), and risk score from the ARlncRNA model (HR = 1.014; 95% CI, 1.005−1.022, p = 0.002) were independent predictors (**Figures 6A, B**). Moreover, the risk score was a risk factor, as the 5-year survival rate was lower if the risk score was >0.943.

### 3.5 Correlation Between the Model Risk Score and Clinical Data

We performed stratified analysis based on age, sex, clinical stage, T stage, M stage, and N stage. The mean risk score for each factor was compared between the two groups of patients. **Table 2** shows that the risk score from the ARlncRNA-based model was unrelated to sex (p = 0.204), age (≤65 vs. > 65 years old, p = 0.512), clinical stage (stage I–II vs. stage III–IV, p = 0.623), M stage (M0 vs. M1, p = 0.379), and N stage (N0 vs. N1–2, p = 0.556). The risk score for patients with T3–T4 stage was significantly higher than that for patients with T1–T2 stage (p = 0.005).

**Table 2:** Correlation analysis between risk scores valued by new prediction model and clinicopathologic parameters.

Clinicopathological parameters	Group	N	Mean	SD	t	p-value
Age	≤65	202	3.637	19.871	0.657	0.512
	>65	270	2.687	6.133		
Gender	Female	222	2.281	4.082	−1.274	0.204
	Male	250	3.815	18.544		
Clinical stage	Stage I–II	273	2.855	17.139	−0.492	0.623
	Stage III–IV	199	3.421	6.982		
T stage	T1–2	94	1.277	1.658	−2.806	0.005
	T3–4	378	3.545	15.36		
M stage	M0	396	2.953	14.856	−0.882	0.379
	M1	76	3.823	5.612		
N stage	N0	282	2.825	16.87	−0.59	0.556
	N1–2	190	3.491	7.115		

N, number; SD, standard deviation.

### 3.6 Signaling Pathways Enriched With the Nine ARlncRNAs

**Figure 7:**
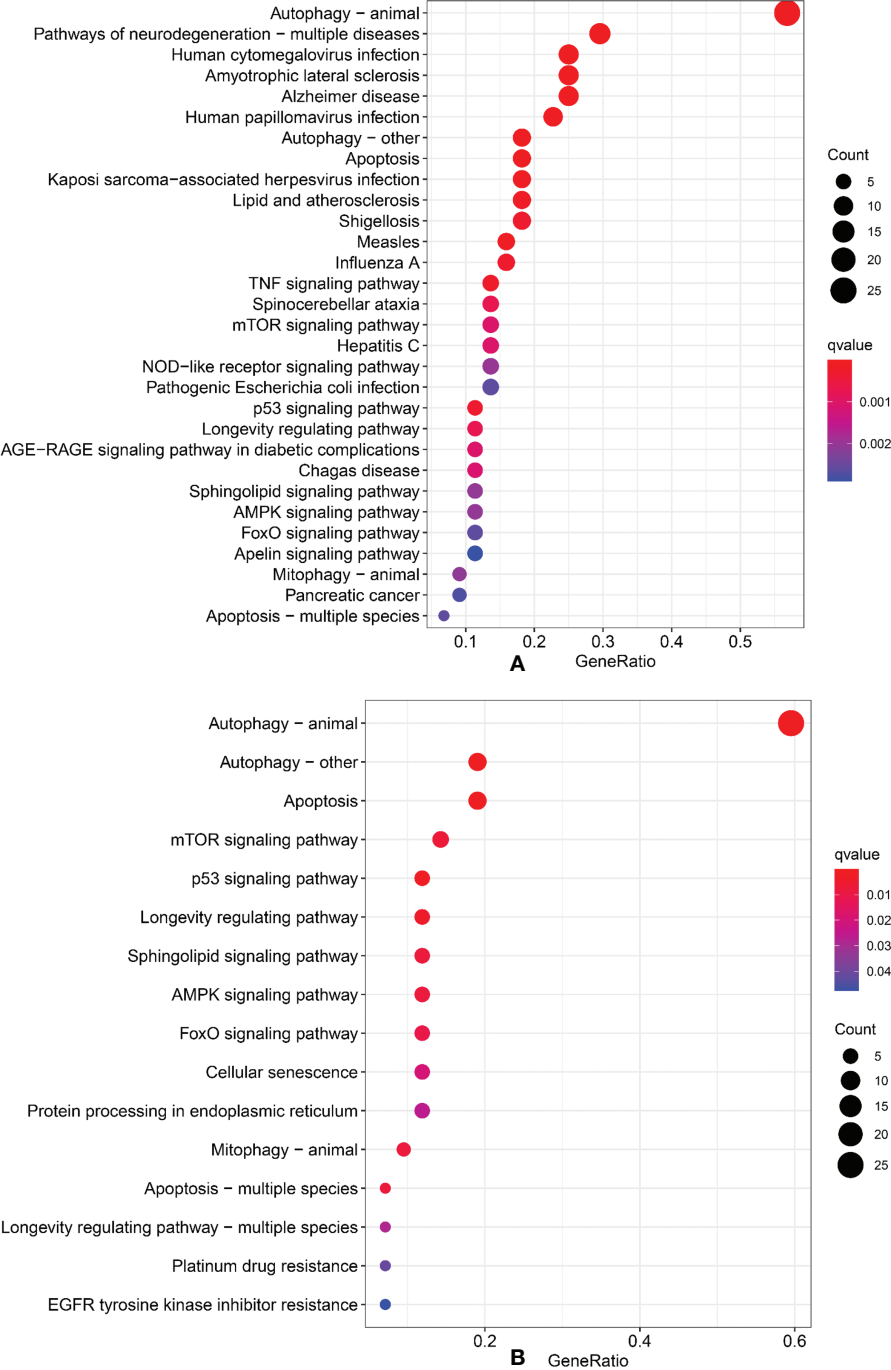
Gene Ontology and Kyoto Encyclopedia of Genes and Genomes. **(A)** GO analysis. **(B)** KEGG analysis.

We performed Gene Ontology (GO) and Kyoto Encyclopedia of Genes and Genomes (KEGG) to identify the signal pathways of enrichment of genes that construct an interaction network with lncRNAs in the model. The results showed that those genes were most enrichment in autophagy signal pathways (**Figures 7A, B**).

### 3.7 Validation *In Vitro*

**Figure 8:**
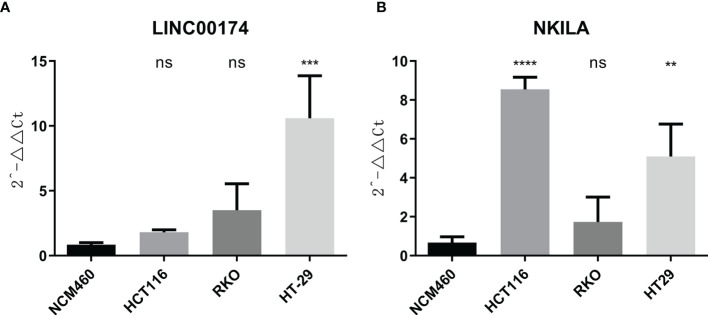
The expression of *LINC00174* and *NKILA* was detected by qRT-PCR. **(A)** The expression of *LINC00174* among normal colonic epithelial cell line and colorectal cancer cell lines. **(B)** The expression of *NKILA* among normal colonic epithelial cell line and colorectal cancer cell lines. All p-values were the comparison of the lncRNA expression of HCT116, RKO, HT29, and that of NCM460. ****p < 0.0001; ***0.0001 < p < 0.001; **0.01 < p < 0.001; ns: p > 0.05.

**Figure 9:**
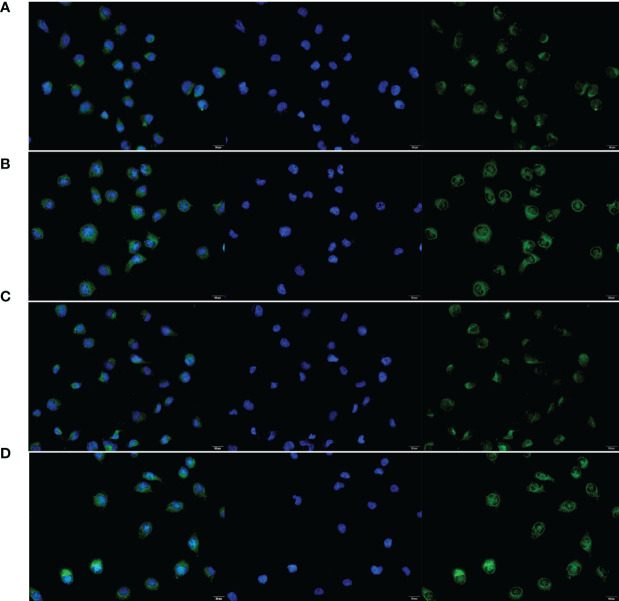
The subcellular localization of *LINC00174* and *NKILA* between NCM460 and HT29 cells. **(A)** Subcellular localization of *LINC00174* in NCM460 cells. **(B)** Subcellular localization of *LINC00174* in HT29 cells. **(C)** Subcellular localization of *NKILA* in NCM460 cells. **(D)** Subcellular localization of *NKILA* in HT29 cells.

**Figure 10:**
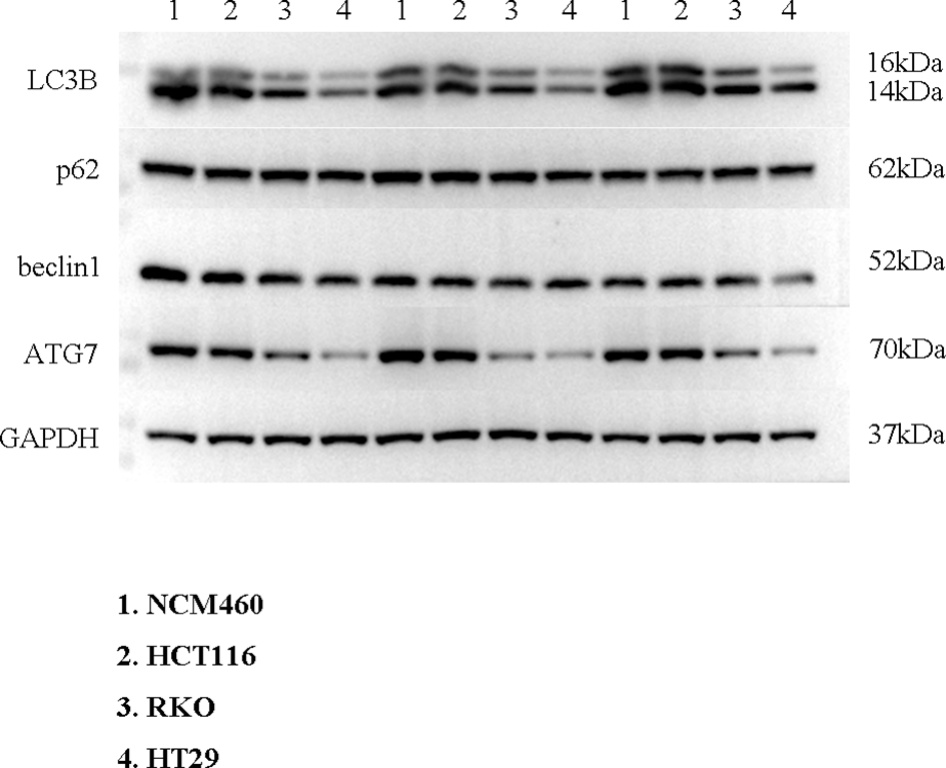
The protein expression of autophagy-related genes was detected by Western blot.

**Figure 11:**
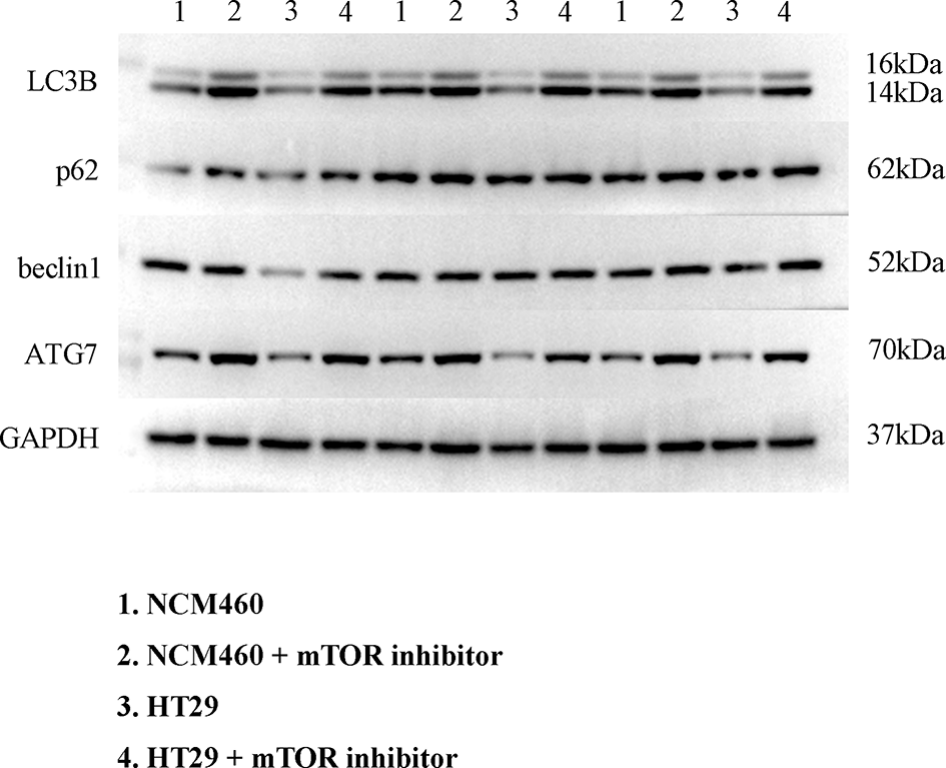
Expression of autophagy-related proteins after autophagy activation.

**Figure 12:**
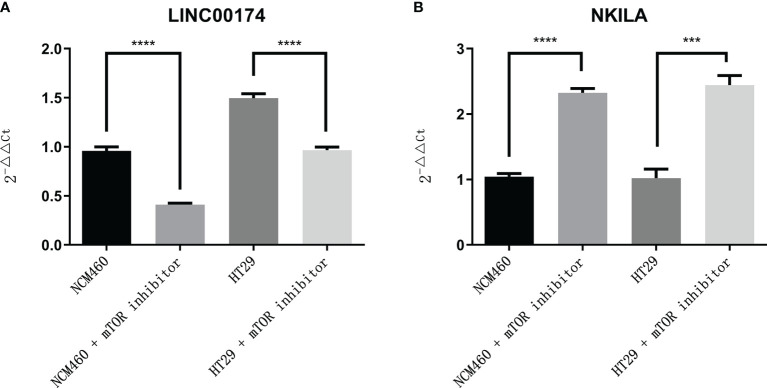
The expression of *LINC00174* and *NKILA* after autophagy activation. **(A)** *LINC00174*, **(B)** *NIKLA*. ****p < 0.0001; ***0.0001 < p < 0.001.

To verify the expression of selected lncRNAs, i.e., *NKILA* and *LINC00174*, in colorectal cancer cell lines, qRT-PCR was performed. As shown in results, *NKILA* and *LINC00174* were all higher expressed in colorectal cancer cell line HT29 compared to normal colonic epithelial cell line NCM460 (**Figure 8**). FISH assay was used to detect the subcellular localization of *LINC00174* and *NKILA*. The results showed that the target genes *LINC00174* and *NKILA* were both expressed in the nucleus and cytoplasm of the NCM460 and HT29 cells (**Figure 9**). In addition, the protein expression of autophagy-related genes was detected by Western blot. The results showed that, compared to NCM460 cell, *LC3B* and *ATG7* were lower expressed in RKO and HT29 cell (**Figure 10**). To detect the relation of *NKILA* and *LINC00174* with autophagy, we stimulated the cells with mTOR inhibitor to activate the autophagy. As shown in the results, significant upregulation of *LC3B* and *ATG7* protein expression was observed between HT29 and NCM460, which suggested that they were related to the activation of autophagy (**Figure 11**). In addition, compared with normal cultured cells, the expression of *LINC00174* was significantly decreased in both NCM460 and HT29 cells after the addition of mTOR inhibitor, while the expression of *NKILA* was significantly increased (**Figure 12**), which suggested that *NKILA* and *LINC00174* were related to autophagy.

## 4 Discussion

Cell death occurs *via* necrosis, apoptosis, and autophagy. Cell apoptosis and autophagy are programmed cell death pathways, and current studies show that apoptosis and autophagy are both regulated by lncRNAs and that regulating the expression level of lncRNAs involved in apoptosis and autophagy regulates the biological behavior of tumor cells. Wei et al. (23) showed that, in CRC, lncRNA *CA3-AS1* reduces *miR-93* expression by directly binding to *miR-93*, thereby promoting CRC apoptosis and suppressing tumor proliferation and invasion. In another study, lncRNA *FAL1* promoted CRC apoptosis, thereby reducing tumor proliferation (24).

ARlncRNAs and apoptosis-related lncRNAs may have similar effects. In recent years, researchers have shown increased interest in the role of lncRNAs in tumor autophagy. lncRNA *SLCO4A1-AS1* binds to *miR-508-3p* to upregulate *PARD3*, thereby promoting protective CRC autophagy and CRC proliferation (25). Zheng et al. (26) showed that the survival time of CRC patients is shorter for those with high lncRNA *HAGLROS* expression than for those with low expression. *HAGLROS* inhibits apoptosis and promotes autophagy to regulate tumor biological behavior mainly *via* the *PI3K/AKT/mTOR* and *miR-100/ATG5* pathways. These data indicate that apoptosis and autophagy may have common regulatory pathways. We suspect that *HAGLROS* may be a marker for predicting the prognosis of patients with CRC. Another study showed that lncRNA *Malat1* directly binds to *miR-101* to regulate CRC autophagy, apoptosis, and proliferation. Further studies showed that the use of 3-methyladenine, an autophagy inhibitor, decreased *Malat1*-induced cell proliferation and promoted *Malat1*-induced apoptosis (27). Taken together, these studies show that lncRNAs play important roles in activating CRC autophagy, suggesting that lncRNAs regulate tumor biological behavior by activating CRC autophagy pathways, thereby enhancing tumor cell proliferation. This conclusion should be taken into consideration in clinical treatment.

In addition, ARlncRNAs play important roles in resistance to chemotherapy. Liu et al. (28) showed that lncRNA *nuclear paraspeckle assembly transcript 1* (*NEAT1*) is highly expressed in CRC tissues and cell lines and is negatively correlated with *miR-34a*, which is involved in autophagy activation *via* targeted sites (*HMGB1*, *ATG9A*, and *ATG4B*). *NEAT1* knockdown significantly inhibits CRC proliferation and enhances sensitivity to 5-fluorouracil (5-FU). Wang et al. (29) showed that lncRNA *H19* triggers autophagy and induces 5-FU resistance in CRC cells *via* the *miR-194-5p/SIRT1* pathway. *H19* expression is significantly increased in patients with recurrent CRC, and the recurrence-free survival time is significantly shorter in patients with high *H19* expression than in patients with low *H19* expression. Han et al. (30) showed that, in CRC, the expression levels of lncRNA *SNHG14* and *ATG14*, an autophagy-related gene, are significantly increased and that lncRNA *SNHG14* regulates tumor proliferation, invasion, migration, apoptosis, and autophagy *via* the *miR-186/ATG14* axis. High *ATG14* expression significantly promotes the proliferation and reduces the apoptosis of cisplatin-resistant CRC cell lines. The expression of autophagy-related *LC3B* decreases after lncRNA *SNHG14* knockdown in cisplatin-resistant CRC cell lines. These studies provide direct evidence that lncRNAs are closely related to autophagy and regulate drug resistance of CRC cells *via* autophagy pathways. ARlncRNAs may become novel treatment targets for resistance reversal.

Numerous studies have shown that lncRNA-based models can predict the prognosis of CRC patients (10–12). However, no ARlncRNA-based model has been established for predicting the prognosis of CRC patients. One study showed that an ARlncRNA-based model can predict the prognosis of patients with lung adenocarcinoma (20). In this study, we established an ARlncRNA-based model to predict the survival of CRC patients for the first time. We performed univariate analysis and obtained 32 prognostic ARlncRNAs from 612 tissue samples from TCGA and then performed multivariate Cox regression analysis to build a risk score model of nine significant prognostic ARlncRNAs (*NKILA*, *LINC00174*, *AC008760.1*, *LINC02041*, *PCAT6*, *AC156455.1*, *LINC01503*, *LINC00957*, and CD27-AS1). Next, we calculated the risk score for each CRC patient based on this model. Survival analysis showed that the 5-year OS was significantly higher in the low-risk group (risk score < 0.943) than in the high-risk group. A multivariate ROC curve showed that the model had high sensitivity and accuracy in predicting the 5-year OS rate (AUC = 0.717). Moreover, the risk score from this model, based on nine ARlncRNAs, was an independent predictor of CRC.

*LINC01503* is an ARlncRNA that we screened that is negatively correlated with patient prognosis. Previous studies have shown that *LINC01503* is highly expressed in CRC cell lines. *LINC01503* overexpression promotes CRC proliferation and invasion, while *LINC01503* silencing inhibits CRC proliferation and invasion. Moreover, *LINC01503* regulates CRC proliferation and invasion *via* the *miR-4492/FOXK1* signaling pathway (31). In gastric cancer and brain glioma, *LINC01503* promotes tumor proliferation and invasion *via* the Wnt signaling pathway (32, 33). Using bioinformatics analysis, we concluded that *LINC01503* is an autophagy-related prognostic predictor for CRC, but further research is needed to investigate the mechanism by which *LINC01503* regulates tumor biological behaviors *via* autophagy pathways.

Numerous studies have shown that *PCAT6* also plays an important role in tumor proliferation and invasion, such as in non-small cell lung cancer (34–36), breast cancer (37), cervical cancer (38, 39), and liver cancer (40, 41). Furthermore, *PCAT6*, a lncRNA, is highly expressed in colon cancer and is closely related to tumor malignancy. High *PCAT6* expression is associated with low survival. *PCAT6* activates the expression of antiapoptotic *ARC* and inhibits colon cancer cell apoptosis by increasing *EZH2* expression (42). This study showed that *PCAT6* is negatively correlated with the prognosis of CRC patients. Moreover, this study showed that *PCAT6* is associated with autophagy and that *PCAT6* probably increases tumor malignancy *via* autophagy pathways. Therefore, *PCAT6* may regulate tumor biological behaviors through different molecular mechanisms, but research on autophagy pathways is still lacking. Another study showed that *PCAT6* is an adverse prognostic predictor of CRC. Moreover, *PCAT6* inhibits *miR-204* expression, thereby promoting activation of the *HMGA2/PI3K* pathway and enhancing 5-FU resistance in CRC (43). Further research is needed to investigate additional mechanisms through which *PCAT6* affects the biological behaviors of CRC.

Colorectal cancer patients with overexpression of *LINC00174* have a poor prognosis. Overexpression of *LINC00174* promotes the proliferation, migration, and invasion of colorectal cancer cells by regulating *Mir-1910-3p/TAZ* and *Mir-3127-5p/E2F7* signaling pathways (44, 45). *LINC00174*, as an autophagy-related lncRNA, secreted by vascular endothelial cells, inhibits autophagy by *SRSF1/P53* signaling pathway, which can alleviate myocardial insulin–reperfusion injury (46). In addition, *NKILA* can enhance autophagy of *HK2* cells through *Mir-140-5p/CLDN2/LPS* pathway to induce acute kidney injury (47). In our research, we found that *NKILA* and *LINC00174* were related to autophagy in colorectal cancer. However, at present, no study has found whether *LINC00174* and *NKILA* can affect the occurrence and development of colorectal cancer through autophagy, which will also be the focus of our follow-up research.

## 5 Conclusion

We constructed a novel prediction model based on nine ARlncRNAs to predict the prognosis of CRC patients. Moreover, we verified the relationship among *LINC00174*, *NKILA*, and autophagy in colorectal cancer cells. Our results provide new ideas for further research on potential mechanisms involved in regulating the biological behaviors of CRC.

### 5.1 Limitations

The prediction model based on nine ARlncRNAs has some limitations. First, no prospective studies have been conducted to confirm its reliability. Second, while we plotted the complex interactive networks between ARlncRNAs and mRNAs, we did not perform in-depth pathway research. Last, we did not consider each patient’s treatment plan in this study, which may affect the study results. In summary, few studies have been conducted to investigate the use of ARlncRNAs to predict the prognosis of patients with CRC and how ARlncRNAs regulate autophagy. In the future, well-designed, randomized, controlled trials are needed to validate the reliability of this model.

## Data Availability Statement

Publicly available datasets were analyzed in this study. This data can be found here: The Cancer Genome Atlas (https://portal.gdc.cancer.gov/repository), the Ensembl database (https://asia.ensembl.org/index.html), and the Human Autophagy database (http://www.autophagy.lu/clustering/index.html).

## Ethics Statement

Ethical review and approval was not required for the study on human participants in accordance with the local legislation and institutional requirements. Written informed consent for participation was not required for this study in accordance with the national legislation and the institutional requirements.

## Author Contributions

WX: fundraising. WX, YS, and JD: revision and confirmation of final manuscript. GX and MY: validation *in vitro* and manuscript revision. GX, QW, SZ, and LZhao: data analysis and drafting of manuscript. LZhu, TX, and RC: data collection and collation. CL and QL: optimize and organize pictures. All authors contributed to the article and approved the submitted version.

## Funding

This study was supported by “Ten Thousand Plan” Youth Talent Project in Yunnan Province and Innovation Fund project of Graduate Student in Kunming Medical University (2021S242).

## Conflict of Interest

The authors declare that the research was conducted in the absence of any commercial or financial relationships that could be construed as a potential conflict of interest.

## Publisher’s Note

All claims expressed in this article are solely those of the authors and do not necessarily represent those of their affiliated organizations, or those of the publisher, the editors and the reviewers. Any product that may be evaluated in this article, or claim that may be made by its manufacturer, is not guaranteed or endorsed by the publisher.

## Abbreviations

CRC, colorectal cancer; TNM, tumor–node–metastasis; OS, overall survival; lncRNA, long noncoding RNA; ARlncRNA, autophagy-related long noncoding RNA; HADb, Human Autophagy database; mRNA, messenger RNA; ROC, receiver operating characteristic; AUC, the area under the curve; GO, Gene Ontology; KEGG, Kyoto Encyclopedia of Genes and Genomes; qRT-PCR, quantitative real-time polymerase chain reaction; FISH, fluorescence *in situ* hybridization; mTOR, *mammalian target of rapamycin*; *NEAT19*, nuclear paraspeckle assembly transcript 1; 5-FU, 5-fluorouracil.
