# Supplementary material for: A Novel Prognostic Prediction Model for Colorectal Cancer Based on Nine Autophagy-Related Long Noncoding RNAs
Source: Front Oncol. 2021 Oct 8;11:613949. doi: 10.3389/fonc.2021.613949 (PMC8531750; doi:10.3389/fonc.2021.613949)
Supplement: Supplementary file 1 [file Table_1.docx]

Supplementary Material

# Supplementary Table 1 Antibodies used in Western blot

| **Supplementary Table 2** Antibodies used in Western blot | | | | |
| --- | --- | --- | --- | --- |
| Proteins | Origin | Concentration | Protein size | Sources of species |
| *LC3B* | abcam ab192890 | 1:2000 | 14, 16 KD | rabit |
| *p62* | abcam ab109012 | 1:1000 | 62 KD | rabit |
| *beclin1* | abcam ab207612 | 1:2000 | 52 KD | rabit |
| *ATG7* | abcam ab52472 | 1:5000 | 70 KD | rabit |
| *GAPDH* | abmart P30008M | 1:1000 | 37 KD | rabit |
| secondary antibody | CST 7074 | 1:2000 |  |  |

# Supplementary Table 2 ARlncRNAs related to prognosis in colorectal cancer patients

| **Supplementary Table 3** ARlncRNAs related to prognosis in colorectal cancer patients | | | |  |
| --- | --- | --- | --- | --- |
|  |  |  |  |  |
| ARlncRNAs | HR | 95% CI | *P* value |  |
| *LBX2-AS1* | 2.008 | 1.180-3.417 | 0.01 |  |
| *NKILA* | 1.808 | 1.201-2.722 | 0.005 |  |
| *LINC00174* | 2.05 | 1.085-3.872 | 0.027 |  |
| *AC104667.2* | 1.878 | 1.023-3.448 | 0.042 |  |
| *AC005840.4* | 2.217 | 1.212-4.056 | 0.01 |  |
| *AC008760.1* | 2.825 | 1.398-5.708 | 0.004 |  |
| *AL591845.1* | 1.551 | 1.157-2.078 | 0.003 |  |
| *AC010973.2* | 2.355 | 1.222-4.536 | 0.01 |  |
| *AC009779.2* | 2.45 | 1.368-4.389 | 0.003 |  |
| *AL139384.1* | 1.879 | 1.040-3.396 | 0.037 |  |
| *LINC01063* | 2.046 | 1.045-4.006 | 0.037 |  |
| *AC073611.1* | 2.78 | 1.350-5.724 | 0.006 |  |
| *CD27-AS1* | 2.909 | 1.705-4.960 | <0.001 |  |
| *AC105219.1* | 1.563 | 1.039-2.350 | 0.032 |  |
| *AC027796.4* | 2.16 | 1.215-3.841 | 0.009 |  |
| *LINC02041* | 1.598 | 1.114-2.292 | 0.011 |  |
| *PCAT6* | 2.199 | 1.347-3.590 | 0.002 |  |
| *LINC01836* | 1.808 | 1.231-2.656 | 0.003 |  |
| *AC005261.3* | 2.069 | 1.071-3.998 | 0.03 |  |
| *TMEM9B-AS1* | 1.919 | 1.067-3.450 | 0.029 |  |
| *AC108488.1* | 2.798 | 1.601-4.889 | <0.001 |  |
| *AC040977.1* | 1.774 | 1.065-2.956 | 0.028 |  |
| *AL132712.1* | 1.863 | 1.016-3.417 | 0.044 |  |
| *CH17-340M24.3* | 1.654 | 1.056-2.591 | 0.028 |  |
| *AC156455.1* | 1.688 | 1.150-2.476 | 0.007 |  |
| *LINC01503* | 2.008 | 1.234-3.268 | 0.005 |  |
| *AL451050.2* | 2.198 | 1.071-4.512 | 0.032 |  |
| *AC027307.2* | 2.487 | 1.383-4.470 | 0.002 |  |
| *ZEB1-AS1* | 5.233 | 2.370-11.558 | <0.001 |  |
| *AL162586.1* | 1.938 | 1.133-3.313 | 0.016 |  |
| *LINC00957* | 2.284 | 1.161-4.494 | 0.017 |  |
| *AC023043.1* | 1.779 | 1.125-2.812 | 0.014 |  |
| HR: hazard radio; CI: confidence interval | | | |  |
